# Survival Prediction of Children Undergoing Hematopoietic Stem Cell Transplantation Using Different Machine Learning Classifiers by Performing Chi-Square Test and Hyperparameter Optimization: A Retrospective Analysis

**DOI:** 10.1155/2022/9391136

**Published:** 2022-09-25

**Authors:** Ishrak Jahan Ratul, Ummay Habiba Wani, Mirza Muntasir Nishat, Abdullah Al-Monsur, Abrar Mohammad Ar-Rafi, Fahim Faisal, Mohammad Ridwan Kabir

**Affiliations:** ^1^Department of EEE, Islamic University of Technology, Gazipur, Bangladesh; ^2^Department of CSE, Islamic University of Technology, Gazipur, Bangladesh

## Abstract

Bone marrow transplant (BMT) is an effective surgical treatment for bone marrow-related disorders. However, several associated risk factors can impair long-term survival after BMT. Machine learning (ML) technologies have been proven useful in survival prediction of BMT receivers along with the influences that limit their resilience. In this study, an efficient classification model predicting the survival of children undergoing BMT is presented using a public dataset. Several supervised ML methods were investigated in this regard with an 80-20 train-test split ratio. To ensure prediction with minimal time and resources, only the top 11 out of the 59 dataset features were considered using Chi-square feature selection method. Furthermore, hyperparameter optimization (HPO) using the grid search cross-validation (GSCV) technique was adopted to increase the accuracy of prediction. Four experiments were conducted utilizing a combination of default and optimized hyperparameters on the original and reduced datasets. Our investigation revealed that the top 11 features of HPO had the same prediction accuracy (94.73%) as the entire dataset with default parameters, however, requiring minimal time and resources. Hence, the proposed approach may aid in the development of a computer-aided diagnostic system with satisfactory accuracy and minimal computation time by utilizing medical data records.

## 1. Introduction

Cancer kills millions of people, even in its most curable forms. According to the statistics of 2020, the estimated death tolls in the USA from colon, pancreatic, lung, breast, and prostate cancers are 53200, 47050, 135720, 42690, and 33330, respectively [1]. When there is no cure, physicians endeavor to extend the lifespan of a cancer patient through surgery, radiation therapy, or chemotherapy as alternative methods of cancer treatment [2]. For various reasons, the high dose of medication during chemotherapy or radiation therapy causes bone marrow damage in patients [2]. Bone marrow (BM), a delicate, elastic adipose tissue located inside most skeleton structures, is responsible for creating the red blood cells of human blood [3, 4]. It also contains hematopoietic stem cells (HSC) that are merely immature blood-forming stem cells endowed with idiosyncratic properties like self-renewal, and they form populations of progenitor cells through cell division and differentiation [4–6]. However, the concept of BMT, otherwise known as hematopoietic stem cell transplant (HSCT), gleans from the postulation of eliminating dysfunctional body parts and replacing them with healthy ones [7]. Although it is a life-saving treatment, it has potential life-threatening risks [8]. Clinical HSCT commenced in 1957, at a time when the health domain was inadequately fathomed about HSCs, immunological reactions to transplants, and even about the specification of antigens steering the course of action [9]. HSCT is not a surgery, rather a specialized treatment for people afflicted by specific cancers or certain medical conditions [10, 11]. The target of such a therapy is transfusing functional BM into a patient, subsequent to their own diseased BM being medicated for exterminating the aberrant cells [11]. The three prime objectives of HSCT are (a) replacement of deceased stem cells affected by chemotherapy, (b) replacement of diseased marrow that is impotent to synthesize its endemic progenitor cells, and (c) infusion of allografts to assist in locating and destroying malignant cells [12].

Healthy BM can be either extracted from the patient (autologous transplant) or conferred on by a volunteer donor (allogeneic transplant). In the case of autologous transplant, stem cells come from any other healthy organ of the patient [3]. And for an allogeneic transplant, a donor with closely matched human leukocyte antigens (HLAs) is needed [10]. Most of the times, siblings, having the same parents, make for the closest matches, although other close relatives or perhaps an unrelated patron can also be a successful match. There are two ways of collecting donor stem cells for transplant: (a) BM collection and (b) leukapheresis [13]. When a patient receives highly matched proteins from a donor, the odds of developing a severe adverse reaction, known as graft-versus-host disease (GVHD), are minimized [3]. Given that a donor cannot be found, cord blood transplants (stem cells collected from the umbilical cord), parent-child, and HLA haplotype mismatched transplants (stem cells collected from a parent, child, or sibling) can be performed [3]. HSCT is broadly adopted for hematopoietic system-acquired and congenital illnesses. According to the Health Resources and Services Administration, almost 23 million people have registered with the donor registry. Besides, the donor registry currently contains approximately 305,000 units of cord blood. The National Cord Blood Inventory (NCBI) provides around 112,000 units, which is reflected in this number, with an additional 4,000 units projected to be available in 2020. The Center for International Blood and Marrow Transplant Research (CIBMTR) registered a total of 9,267 related and unrelated BMTs conducted in the United States in 2018 [14]. According to a survey undertaken by UPMC Children's Hospital of Pittsburgh, the percentages of patients who survived 100 or more days after the transplant procedure, the percentage of patients who died of causes other than the underlying disease, and the percentage of patients who survived one or more years after the transplant procedure are 100%, 3%, and 94%, respectively [15].

**Figure 1:**
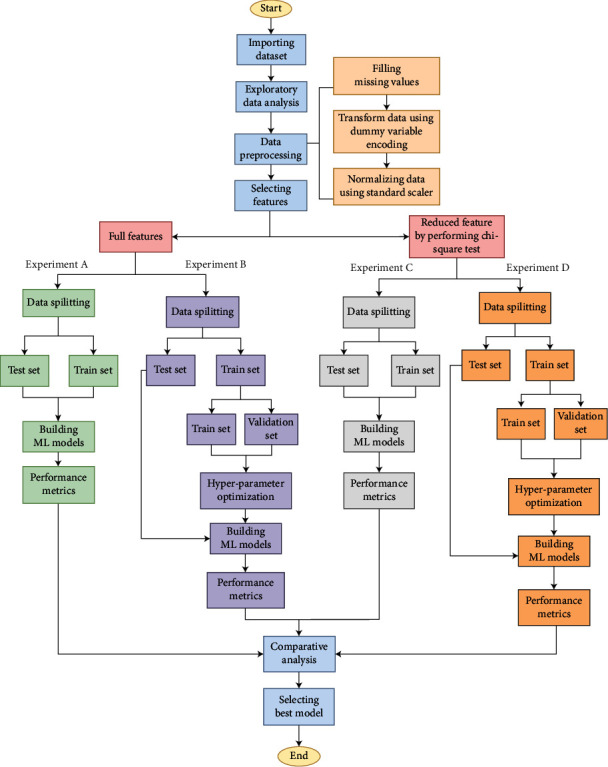
Overall workflow diagram.

To summarize, BMT is a treatment that saves and risks life at the same time. The success rate of BMT is mostly dependent on earlier examination, data collecting, and analysis. Hence, a lot of clinical data collection and generation are required before therapy. In this regard, a machine learning-based support system can help healthcare professionals in a variety of situations. It has significant predictive ability for this type of problem and has been extensively used in recent years in a variety of sophisticated healthcare systems [16–20]. Moreover, it has lately been shown to be incredibly effective in the healthcare arena [21–26]. In the case of BMT, an ML-based support system can play an important role by predicting the patient's survival after treatment and assisting in the necessary preparations prior to therapy. Many previous studies in this domain have been undertaken, but the majority of them have not included the survival prognosis for children undergoing bone marrow transplants utilizing the ML approach. In this type of healthcare scenario, machine learning can be quite useful for prediction. However, the major causes of death in children undergoing HSCT remain unclear. The current BMT method does not allow healthcare providers to determine survivability in advance. It will be very useful for them if a prior prognosis can be established, and they may take necessary actions to provide treatment based on this. The goal of this research is to develop a trustworthy ML-based clinical support system for healthcare professionals involved in the treatment of BMT. In this study, the survival prediction of children who received BMT was thoroughly investigated using seven supervised ML classifiers, such as decision tree (DT), random forest (RF), logistic regression (LR), *K*-nearest neighbors (KNN), gradient boosting classifier (GBC), AdaBoost (AdB), XG Boost (XGB), and a dataset obtained from the UCI ML repository [27]. The Chi-squared feature ranking technique was deployed after preprocessing the dataset to discover the important factors of survivability [28]. The entire study consists of four experiments, such as that (A) with a full set of features and default hyperparameters, (B) with a full set of features and HPO, (C) with a reduced feature dataset (based on the Chi-squared test) and default hyperparameters, and (d) with a reduced feature dataset (based on the Chi-squared test) and HPO followed by a rigorous quantitative and qualitative analysis. An overall workflow diagram of this work is depicted in Figure 1.

The contributions of this study may be summarized as follows: (1) development of a suitable predictive model from raw data, (2) determination of critical factors influencing post-BMT survival, and (3) improvement of the prediction accuracy by reducing dimensionality problems. To the best of our knowledge, this dataset has never been exploited and analyzed in this way before, and it may appear as a significant contribution in helping the healthcare industry to develop a more trustworthy e-healthcare system and create a new horizon in the medical sector.

## 2. Literature Review

In recent times, ML techniques have been extensively exploited for diagnosis, prognosis, and therapeutics in the healthcare sector. Its applications are not limited to treatment procedures; rather, they are expeditiously gaining traction in a variety of research fields. In a narrative review, Nathan et al. highlighted essential ML concepts for novice readers, discussed the applicability of ML in hematology-related malignancies, and indicated key points for practitioners to consider before evaluating ML studies [29]. Vibhuti et al. also conducted a comparative evaluation of ML methods utilized in the discipline of HSCT, examining the categories of data flows incorporated, designated ML algorithms used, and therapeutic consequences monitored [30]. On the other hand, patients with acute leukemia (AL) undergoing HSCT from unrelated donors exhibit a plethora of variations, even after rigorous genetic matching. To address this, Ljubomir et al. sought to develop an algorithm to predict the five-year survival of patients' postallogeneic transplant [31]. Similarly, Brent et al. trained a Bayesian ML model to predict acute GVHD, including mortality by day 180 [32]. However, with better donor data collection, it is possible to generate a more precise approximation of individual donor availability, as estimating group averages for the distinct donors is an untrustworthy proposition. As a solution to this problem, Adarsh et al. suggested an ML-based technique for estimating the availability of each listed donor and validation of forecasting accuracy [33]. Additionally, Li et al. focused on creating and verifying an ML technique for estimating donor availability, implementing and comparing three ML algorithms [34]. As a result of organized registry establishment and biological data incorporation, data procured from HSCT institutions is becoming highly proliferated and labyrinthine. Consequently, conventional statistical methods are confirmed to be obsolescent. In its provision, Shouval et al. aimed to advocate the implementation of ML and data mining (DM) schemes in the study of HSCT, covering transplant performance prognosis as well as donor selection [35]. Similarly, Jan-Niklas et al. explored current ML breakthroughs in the acute myeloid leukemia (AML) diagnosis as a prototype condition encompassing hematologic neoplasms [36]. Furthermore, the goal of the research by Liyan et al. was to shape an ALL- (acute lymphocytic leukemia-) relapse detection scheme relying on ML methods [37]. In addition, using alternating data tree (ADTree), Kyoko et al. endeavored to design a model for predicting leukemia recidivism within a year following transplantation [38]. For contemplative and prospective analysis, ADTree was also employed by Yasuyuki et al. to scan databases containing information about adult patients with HSCT in Japan [39]. Daniela et al. also examined the organic phenomena associated with self-regeneration and augmentation of hormone-sensitive prostate cancer (known as CD34+ cells) in stable conditions and subsequent transplantation [40]. Moreover, a DM analysis involving 28,236 registered adult HSCT receiving patients from the European Group for Blood and Marrow Transplantation's registry was done by Shouval et al. to predict 100-day overall and nonrelapse mortality, free of leukemia, and 2-year overall survival. The ADTree algorithm was employed to create models using 70% of the data set, and the remaining 30% of the data was utilized to validate them [41]. Moreover, Arabyarmohammadi et al. used the Cox regression model to estimate the probability of patient relapse after acute myeloid leukemia posthematopoietic cell transplantation [42]. Similarly, Iwasaki et al. created a stacked ensemble of the Cox proportional hazard (Cox-PH) regression and 7 machine learning algorithms and discovered prediction accuracy with a *C*-index of 0.670 utilizing the ensemble model [43]. On the other hand, Morvant et al. used machine learning (support vector machine (SVC) and Ridge logistic regression (LR Ridge)) with leave-one-out cross-validation to compare several combinations for predicting bone marrow minimal residual disease (MRD) before autologous stem cell transplant consolidation (ASCT) and discovered AUCs of up to 0.63 and 0.82 for negative vs. positive MRD in the lesion with the highest uptake [44]. Inspired by prior studies proposed earlier and mentioned above, this research attempts to construct a trustworthy clinical support system using supervised ML algorithms and the Chi-square test. To the best of our knowledge, no earlier research has been undertaken to predict children undergoing BMT survivability utilizing the Chi-squared algorithm in conjunction with supervised ML algorithms and HPO. Furthermore, most past research has not focused on establishing a clinical support system that can predict with greater accuracy and includes feature ranking.

In this study, an ML stratagem was adopted for eliciting a prediction of the survival rate of patients who had BMT or HSCT. All previous works augmented the prediction study and related investigation through distinctive strategies; however, all have limitations that need to be overcome. The sole purpose of this research is to investigate whether HPO along with a reduced feature set can provide a reliable outcome using an investigative ML approach and to distinguish the most impactful factors on children's survival who have received BMTs. A preprint has previously been published in [42].

## 3. Methodology

### 3.1. Dataset Description

The dataset used in this study was retrieved from the ML repository at the University of California, Irvine, and the version utilized in this study was extracted from [27]. It covers medical information for children who have been diagnosed with a variety of hematologic diseases and who underwent unmodified allogeneic unrelated donor HSCT [43]. Hence, this dataset comprises 187 occurrences and 37 attributes that contain information about individuals who have been diagnosed with a range of hematologic, malignant, or benign diseases. Most of the attributes contain categorical data, while others contain Boolean and numerical values. The dataset's attributes are listed in Supplementary Materials (Appendix I). Following data extraction, it was subjected to exploratory data analysis using *Jupiter Notebook* and *Python* to determine the dataset's properties.

### 3.2. Chi-Square Test

As a type of statistical procedure, Chi-square tests are used to determine the level of independence between categorical variables. It is also a widely used nonparametric method for parametric and normal distribution testing of nominal data [44]. This technique is intended for feature tests that are independent of one another. This produces the Chi-square score, which is used to identify the most highly correlated feature for ML models to predict desired outcomes [45]. The Chi-square score indicates the degree to which the attributes of a dataset are related. An attribute with a low score indicates that it has a very low predictive ability for the dataset's desired outcome column. Therefore, by utilizing this information, the most critical features may be identified, and more efficient models may be deployed on large datasets. The Chi-squared statistical test formula can be written as follows:

(1) $$\begin{array}{c} \chi^{2}=\sum \frac{{\left(O-E\right)}^{2}}{E},\text{where}\left\{\begin{array}{c} O\text{ denotes the observed frequencies}\\ E\text{ denotes the expected frequencies}. \end{array}\right. \end{array}$$

After preprocessing the data, which includes filling in missing values, encoding categorical variables, and normalization, the Chi-squared statistical test is used to determine the attributes' independence. The top attribute in this list is “PLT recovery,” followed by “ANC recovery,” “time_to_acute_GvHD_III_IV,” “survival_time,” and so on. The summary of the test on this preprocessed dataset is shown in Supplementary Materials (Appendix II).

### 3.3. Hyper Parameter Optimization (HPO)

**Figure 2:**
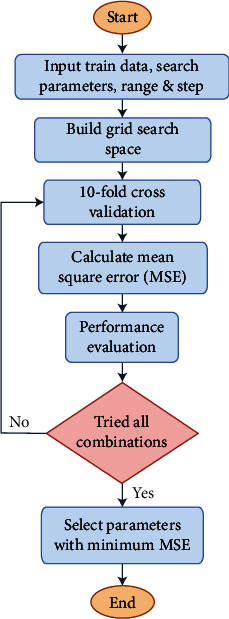
Flowchart of grid search cross-validation (GSCV).

The parameters that define the architecture of ML models are known as hyperparameters. Hence, the optimization of hyperparameters has a substantial impact on the formation of ideal models for certain tasks. While training the model, hyperparameters are optimized using validation data from a dataset. Typically, grid search cross-validation (GSCV) and random search cross-validation (RSCV) are two HPO processes that work well for a variety of ML tasks [46]. HPO is critical for determining the optimal performance of any ML model because it establishes the model's core architecture [47]. Moreover, the importance of HPO was discovered by several researchers and is now widely employed in ML-based prediction [48]. The GSCV evaluates all possible combinations from a given set of hyperparameters, whereas the random search algorithm just attempts some random possible combinations [49]. As a result, even though it takes a bit longer than a random search, the grid search technique yields better results when tuning the hyperparameters of any ML algorithm. Hence, the grid search technique is employed in this study to fine-tune the hyperparameters and achieve better results.  Figure 2 illustrates the workflow of GSCV.

### 3.4. Workflow

**Figure 3:**
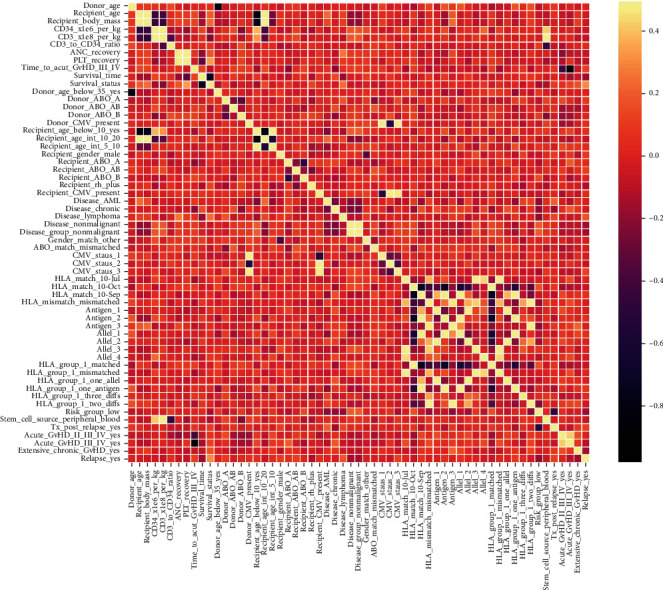
Correlation heatmap (full-feature dataset).

**Figure 4:**
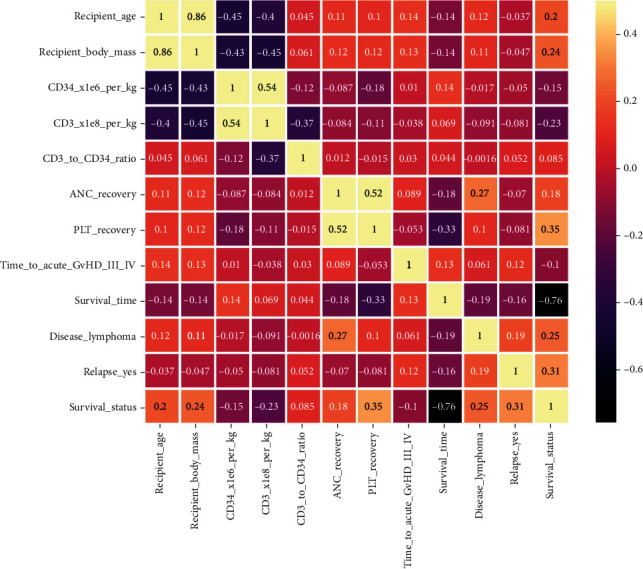
Correlation heatmap (reduced feature dataset).

Following early data analysis, the dataset underwent multiple preprocessing stages before being used in the machine learning models. First, the dataset underwent multiple preprocessing stages before being used in ML models. The missing values of the dataset were filled with mean values for numerical ones and the most frequent values for categorical ones. Since categorical data cannot be handled by ML models, the categorical variables were encoded into a numerical form. The dummy variable encoding technique was employed for this purpose, and the attributes were turned into Boolean attributes that could readily fit into any ML model [50]. Second, the attributes were then normalized using the standard scaling method to avoid bias from the ML models [51], leaving the dataset with 59 columns after preprocessing. To discover the correlation between attributes, the correlation heatmap is generated using the processed dataset, as depicted in Figure 3. Third, the dataset was split into train and test sets in proportions of 80% and 20%, respectively. Seven ML algorithms, DT, RF, LR, KNN, GBC, AdB, and XGB, were fed and trained on this dataset, and performance metrics were obtained. Moreover, the Chi-squared statistical test is used to determine the most important features, and the test score is represented in Supplementary Materials (Appendix II). Once the Chi-squared score is calculated, a minimum number of features are determined that can still predict survival reliably, using fewer electronic health records and computational resources. As a result, the top 11 features were chosen empirically from Supplementary Materials (Appendix II) and were analyzed for the prediction of the models. The correlation heatmap using these 11 features is shown in Figure 4.

As mentioned earlier, a total of four distinct experiments, A, B, C, and D, were carried out in this study. In experiments A and C, no HPO was performed. However, in experiments B and D, the train dataset was cross-validated using GSCV to determine the optimum hyperparameters of the ML model. After training the ML models, the test dataset was fed to evaluate the performance of various models. Finally, all performance metrics were calculated, and various comparisons and analyses were performed to determine the impact of hyperparameter tuning and the use of the full-feature dataset and the reduced dataset, in which attributes were chosen based on the results of the Chi-squared test. This research was entirely carried out on an Intel Core i5-8300H CPU operating at 2.30 GHz, 8 GB of RAM, and an NVIDIA GTX 1050 Ti graphics unit with 4 GB of GPU memory using Jupyter Notebook v6.1.4 (Python 3 v3.8.5) and Anaconda-v4.10.3.

## 4. Results

### 4.1. Experiment A: With a Full Set of Features and Default Hyperparameters

**Figure 5:**
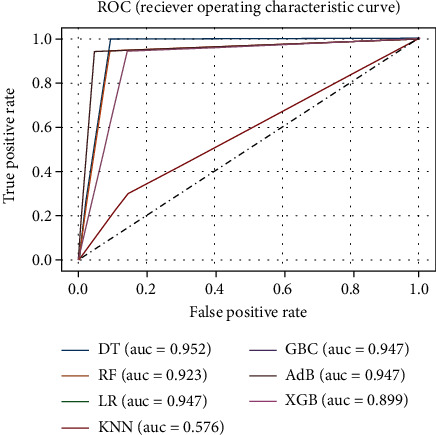
ROC curve for the ML models of experiment A.

This experiment was conducted using the processed full-feature dataset with no optimization of model hyperparameters. The dataset for this experiment has 58 attributes and 1 objective attribute. Figure 3 shows the correlation heatmap for the whole feature dataset. The performance metrics (accuracy, precision, recall, *F*1, and ROC_AUC values) of the ML models are summarized in Table 1. The receiver operating characteristics (ROC) curve for this experiment, as shown in Figure 5, can be used to discover the ideal ML model, thus removing suboptimal models. It can be observed from Table 1 that the models DT, LR, GBC, and AdB have the best accuracy, precision, and *F*1 score, and DT has the highest recall and ROC_AUC.

### 4.2. Experiment B: With a Full Set of Features and Hyperparameter Optimization (HPO)

**Figure 6:**
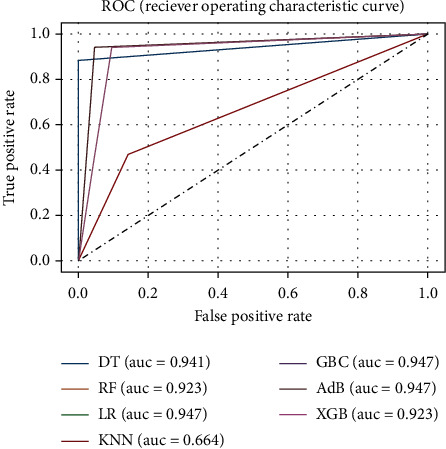
ROC curve for the ML models of experiment B.

In this experiment, the full set of features of the dataset was utilized along with HPO. The training dataset was cross-validated 10-folds using GSCV to determine the optimal hyperparameters and using which all other performance metrics were assessed. The performance metrics of the ML models are summarized in Table 2, and the corresponding ROC curve is depicted in Figure 6. As seen from this table, the algorithms, DT, LR, GBC, and AdB perform reasonably well in this experiment. Moreover, DT outperforms the other algorithms in terms of precision and *F*1 score, whereas LR, GBC, and AdB have the highest recall and ROC_AUC.

### 4.3. Experiment C: A Reduced Dataset Based on Chi-Square Test and Default Hyperparameters

**Figure 7:**
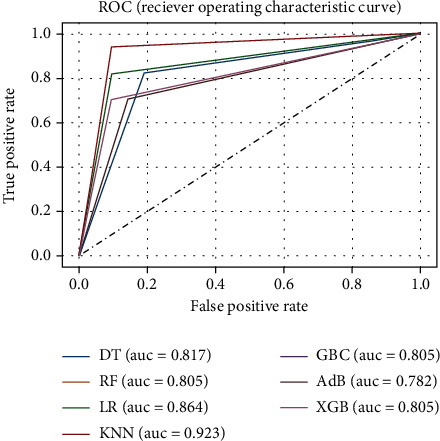
ROC curve for the ML models of experiment C.

This experiment was conducted considering the top 11 features of the dataset, obtained from the Chi-square test. The ML models were initially trained on the selected training set and subsequently verified on the test set using the default hyperparameters without any sort of optimization. The performance metrics are reported in Table 3, and the corresponding ROC curve is presented in Figure 7. It is apparent from Table 3 that KNN surpasses the rest of the classifiers in terms of accuracy, *F*1 score, recall, and the ROC_AUC value. However, in regard to precision, RF, LR, KNN, GNB, and XGB all perform the same.

### 4.4. Experiment D: A Reduced Dataset Based on Chi-Squared Test and Hyperparameter Optimization (HPO)

**Figure 8:**
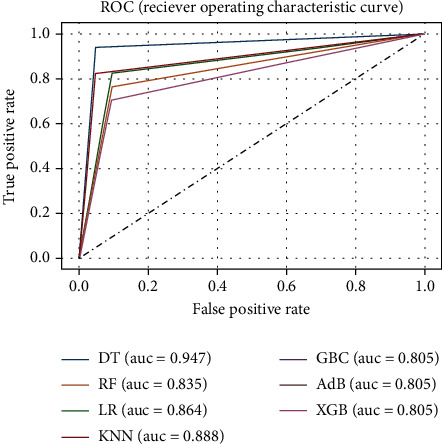
ROC curve for the ML models of experiment D.

Similar to experiment C, experiment D is conducted using the reduced feature dataset with HPO. As before, HPO was performed using GSCV with 10-fold cross-validation to determine the optimal hyperparameters. The reduced dataset comprised of 11 attributes, and the performances were evaluated based on them. The performance metrics are reported in Table 4, and the corresponding ROC curve is presented in Figure 8. From Table 4, it is evident that DT outperforms all other algorithms in every performance metric. However, in terms of precision, DT and KNN perform the best altogether.

## 5. Discussion

**Figure 9:**
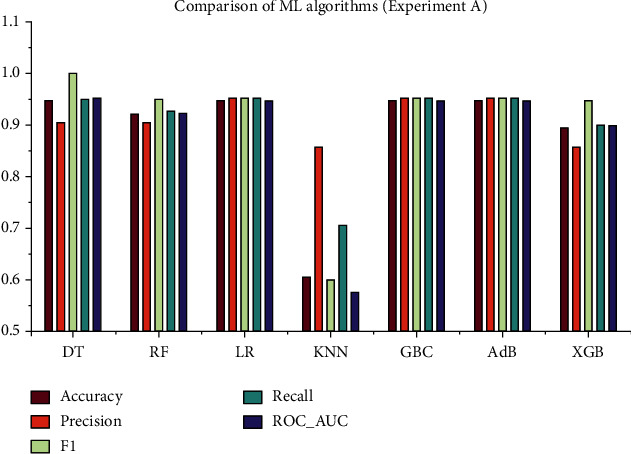
Comparative analysis of the performance metrics for experiment A.

**Figure 10:**
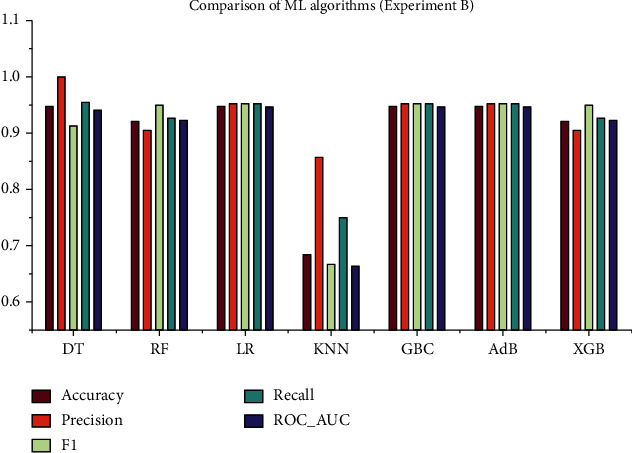
Comparative analysis of the performance metrics for experiment B.

**Figure 11:**
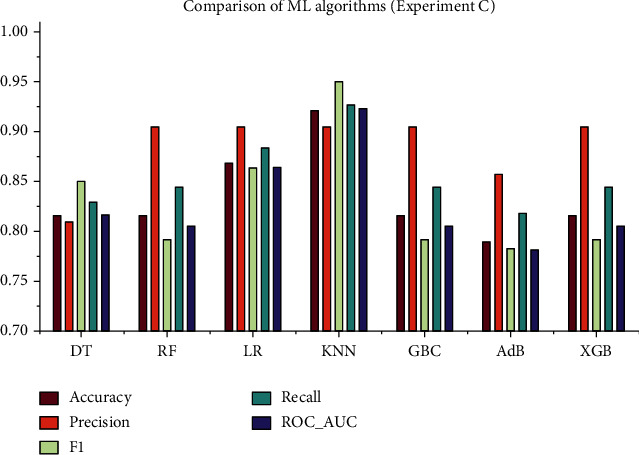
Comparative analysis of the performance metrics for experiment C.

**Figure 12:**
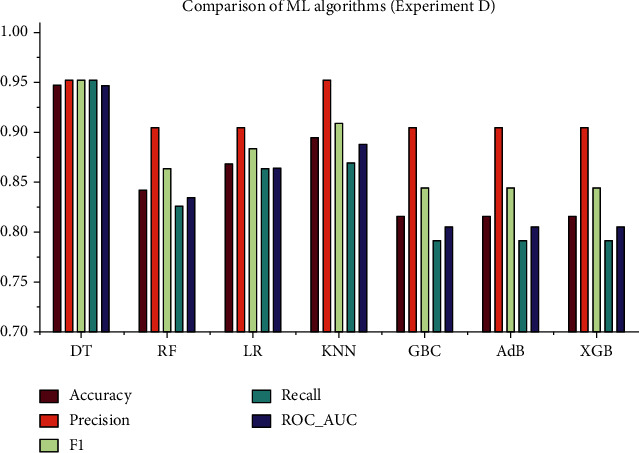
Comparative analysis of the performance metrics for experiment D.

As can be seen, the overall study included four experiments with four different approaches. The whole feature dataset was employed in the experiment A without HPO, and the maximum accuracy was found to be 94.73%, as were the precision (0.9523), recall (1), *F*1 score (0.9523), and ROC_AUC (0.953). In terms of accuracy, the best algorithms are DT, LR, GBC, and AdB. However, in experiment B, the maximum accuracy was 94.73%, and the precision, recall, *F*1 score, and ROC_AUC were 1, 0.9523, 0.9545, and 0.9467, respectively. The well-performing algorithms were the same as in the experiment A, but the overall performance was improved in this experiment since HPO was performed with the 10-fold GSCV method. On the other hand, the experiments C and D are carried out based on the Chi-square test results. The top 11 features from the dataset were extracted, and this reduced dataset was employed in these tests. In experiment C, the maximum accuracy of 92.1% was obtained for KNN, with precision (0.9047), recall (0.95), *F*1 score (0.9268), and ROC_AUC (0.9229). In experiment D, HPO was performed on the same reduced dataset as in experiment C. This time, the performance of all ML algorithms improved significantly, DT having the best performance measures. The performance measures for DT in experiment D are as follows: accuracy (0.9473), precision (0.9523), recall (0.9523), *F*1 score (0.9523), and ROC_AUC (0.9467). The graphical presentation of the comparative analysis of experiments A, B, C, and D is illustrated in Figures 9–12, respectively.

**Figure 13:**
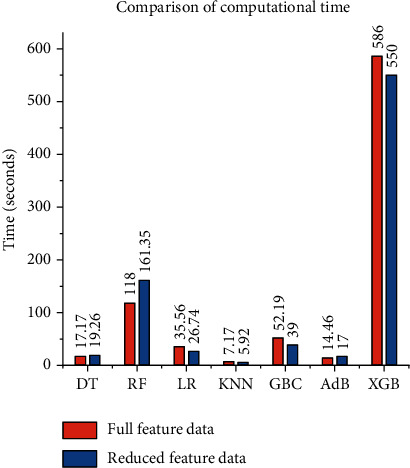
Comparison of computational time (between experiments B and D).

**Figure 14:**
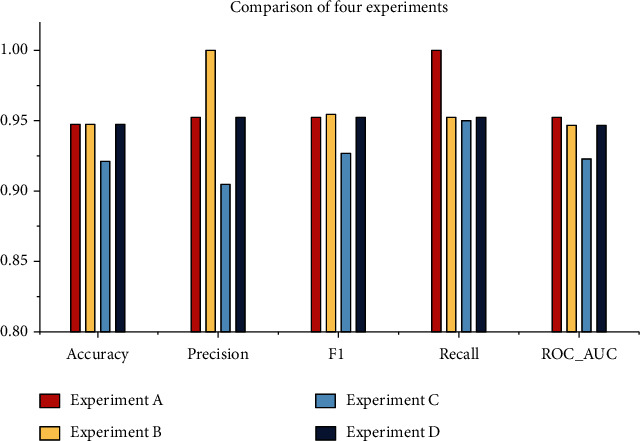
Comparison of performance metrics (experiments A, B, C, and D).

Based on the above four experiments, it is evident that HPO is critical to enhancing the performance of the ML algorithm, and the Chi-square test plays a significant role in determining the most important feature. The computation time of GSCV using complete and reduced feature datasets is shown in Table 5 and is visualized using a bar plot in Figure 13. Most of the ML classifiers required less time in the reduced feature dataset without significantly affecting performance, which is an encouraging result of our study. The comparison between the four experiments is shown in Figure 14 in terms of accuracy, precision, recall, *F*1, and ROC_AUC. The top five critical attributes established in this study are “PLT recovery,” “ANC recovery,” “duration of acute GvHD III IV,” “survival time,” and “recipient body mass.”

Previously, researchers employed a prediction method to predict the survivability of patients receiving BMTs. For instance, Gudys et al. employed a rule-based predictive model in this dataset and produced a tool named RuleKit for predicting BMT survival rates in children [52]. Likewise, Sikora et al. established a framework that is based on decision rules and the rule induction approach [53]. In a similar study, Karami et al. combined ML and feature selection methods to identify the most appropriate factors for predicting AML patient survival [54]. They used six ML algorithms, like DT, RF, LR, naive Bayes, W-Bayes net, and gradient boosted tree (GBT). With an AUC value of 0.930, the GBT was found to have 86.17% accuracy, making it the most accurate predictor of AML patient survival using the relief algorithm for feature selection. Moreover, Leclerc et al. employed a tree-augmented naive Bayesian network to develop a certified decision support tool for selecting the most suitable initial dose of intravenous cyclosporine A (CsA) in pediatric patients undergoing HSCT [55]. A ten-year monocentric dataset was used after discretization using Shannon entropy and equal width intervals. The AUC-ROC of the TAN Bayesian model is 0.804 on average, with a 32.8% misclassification rate and true-positive and false-positive rates of 0.672 and 0.285, respectively. Additionally, Bortnick et al. investigated the outcomes of 65 patients with myelodysplastic syndrome (MDS) in infancy who had received HSCT and had a germline GATA2 mutation (GATA2mut) [56]. Overall survival was found to be 75% after five years, while disease-free survival (DFS) was 70%. On the other hand, Hazar et al. evaluated the results of 62 pediatric patients who received HSCT for relapsed non-Hodgkin lymphoma (rr-NHL). The overall survival (OS) rate was determined to be 65%, whereas the event-free survival (EFS) rate was found to be 48% [57]. However, Qi et al. used the Cox proportional hazard to assess bleeding's independent prognostic value and fine-gray competing risk models for survival analyses, lasso regression to select a training set to derive the bleeding score, and logistic regression to derive the value-added score. There was an increased cumulative incidence of overall mortality (HR = 10.90), nonrelapse mortality (HR = 14.84), and combined endpoints (HR = 9.30), but not the cumulative incidence of relapse in higher bleeding class HSCT patients [58]. The performance comparison of our methodology with some of these state-of-the-art ones is provided in Table 6.

## 6. Conclusion AND Future Works

The bone marrow transplant is a crucial life-saving treatment for a certain type of malignancy. For this reason, early detection of survivability after BMT can play a vital role in the patient's treatment process. Moreover, if healthcare providers have a prior prediction, they can make more informed decisions about treatment options. In this regard, technologies like ML can be useful, since they can be used in situations requiring prediction and can uncover hidden patterns in previous data to create an accurate prediction. Nowadays, it is increasingly being employed in every situation that requires prediction. In this study, we developed a Chi-square feature selection method and an HPO-based efficient model for predicting the survival of children who received BMT and identified the most significant parameters for survival after BMT. All four experiments that were conducted yielded satisfactory predictions. The models operate well on a synthetic dataset that has been constructed from the raw dataset via a series of preprocessing phases that reduce the dataset's dimensionality. On the entire feature synthetic dataset, the experiment A achieves an accuracy of 94.73%. However, as experiment B optimizes the hyperparameters using the same dataset as experiment A, it achieves the highest overall performance of all models. On the other hand, experiments C and D use the 11 most correlated feature dataset based on the Chi-squared test, and experiment D outperforms all performance measures when combined with HPO, achieving high accuracy (94.73%) with less time, data, and resource consumption. In this study, we obtained the maximum accuracy (0.9473), precision (1), recall (1), *F*1 (0.9545), AUC (0.9523), and the top five attributes that influence the survivability rate are “PLT recovery,” “ANC recovery,” “duration of acute GvHD III IV,” “survival time,” and “recipient body mass.” Historically, this dataset has not been evaluated in such a manner before, and it could provide the health sector with a unique perspective. Therefore, this study can make a noteworthy contribution to the development of ML-based healthcare prediction systems in environments where resources are scarce and healthcare practitioners lack more data. The current algorithm performs admirably with our tested dataset and appears to be effective in the clinical phase. This model might be deployed in the clinical phase in the future, and a clinical trial could be done to evaluate and improve the model to make it more robust and trustworthy. To take full advantage of this type of support system, healthcare professionals and patients need be trained on how to use the technology.

## Figures and Tables

**Table 1:** Performance metrics of ML algorithm for experiment A.

Algorithm	Accuracy	Precision	Recall	*F*1	ROC_AUC
DT	**0.9473**	0.9047	**1.000**	0.9500	**0.9523**
RF	0.9210	0.9047	0.9500	0.9268	0.9229
LR	**0.9473**	**0.9523**	0.9523	**0.9523**	0.9467
KNN	0.6052	0.8571	0.6000	0.7058	0.5756
GBC	**0.9473**	**0.9523**	0.9523	**0.9523**	0.9467
AdB	**0.9473**	**0.9523**	0.9523	**0.9523**	0.9467
XGB	0.8947	0.8571	0.9473	0.9000	0.8991

**Table 2:** Performance metrics of ML algorithm for experiment B.

Algorithm	Accuracy	Precision	Recall	*F*1	ROC_AUC
DT	**0.9473**	**1.0000**	0.9130	**0.9545**	0.9411
RF	0.9210	0.9047	0.9500	0.9268	0.9229
LR	**0.9473**	0.9523	**0.9523**	0.9523	**0.9467**
KNN	0.6842	0.8571	0.6666	0.7500	0.6638
GBC	**0.9473**	0.9523	**0.9523**	0.9523	**0.9467**
AdB	**0.9473**	0.9523	**0.9523**	0.9523	**0.9467**
XGB	0.9210	0.9047	0.9500	0.9268	0.9229

**Table 3:** Performance metrics of ML algorithm for experiment C.

Algorithm	Accuracy	Precision	Recall	*F*1	ROC_AUC
DT	0.8157	0.8095	0.8500	0.8292	0.8165
RF	0.8157	**0.9047**	0.7916	0.8444	0.8053
LR	0.8684	**0.9047**	0.8636	0.8837	0.8641
KNN	**0.9210**	**0.9047**	**0.9500**	**0.9268**	**0.9229**
GBC	0.8157	**0.9047**	0.7916	0.8444	0.8053
AdB	0.7894	0.8571	0.7826	0.8181	0.7815
XGB	0.8157	**0.9047**	0.7916	0.8444	0.8053

**Table 4:** Performance metrics of ML algorithm for experiment D.

Algorithm	Accuracy	Precision	Recall	*F*1	ROC_AUC
DT	**0.9473**	**0.9523**	**0.9523**	**0.9523**	**0.9467**
RF	0.8421	0.9047	0.8260	0.8636	0.8347
LR	0.8684	0.9047	0.8636	0.8837	0.8641
KNN	0.8947	**0.9523**	0.8695	0.9090	0.8879
GBC	0.8157	0.9047	0.7916	0.8444	0.8053
AdB	0.8157	0.9047	0.7916	0.8444	0.8053
XGB	0.8157	0.9047	0.7916	0.8444	0.8053

**Table 5:** Comparison of computational time.

Algorithm	Computation time for full-feature data (seconds)	Computation time for reduced feature data (seconds)
DT	17.17	19.26
RF	118.00	161.35
LR	35.56	**26.74**
KNN	7.17	**5.92**
GBC	52.19	**39.00**
AdB	14.46	**17.00**
XGB	586.00	**550.00**

**Table 6:** Performance comparison of our methodology with state-of-the-art ones.

References	Authors	Method	Findings
[55]	K. Karami et al.	ML with feature selection	Accuracy of 85.17% and AUC of 0.930
[56]	V. Leclerc et al.	Tree-augmented naïve Bayesian network	AUC-ROC of 0.804, 32.8% of misclassified patients
[58]	V. Hazar et al.	Kaplan–Meier method and *χ*^2^ test	Overall survival (OS) of 65% and event-free survival (EFS) rate of 48%
[59]	Y. T. Jiaqian Qi et al.	The Cox proportional hazard model and fine-gray competing risk model	Overall mortality (HR = 10.90), nonrelapse mortality (HR = 14.84), and combined endpoints (HR = 9.30)
Proposed study		ML with Chi-squared test	Survival prediction accuracy of 94.73%

## Data Availability

Bone marrow transplant: children dataset from UCI machine learning repository was used in order to support this study and is available at “Bone Marrow Transplant: Children Dataset https://archive.ics.uci.edu/ml/datasets/Bone+marrow+transplant:+children.” This prior study and dataset are cited at relevant places within the text as Ref [27].

## Supplementary Materials

Supplementary Materials The attributes of the dataset are listed in Appendix I, and the summary of the Chi-square test results on the preprocessed dataset is shown in Appendix II.
