# Supplementary material for: Survival Prediction of Children Undergoing Hematopoietic Stem Cell Transplantation Using Different Machine Learning Classifiers by Performing Chi-Square Test and Hyperparameter Optimization: A Retrospective Analysis
Source: Comput Math Methods Med. 2022 Sep 25;2022:9391136. doi: 10.1155/2022/9391136 (PMC9527434; doi:10.1155/2022/9391136)
Supplement: Supplementary Materials — The attributes of the dataset are listed in Appendix I, and the summary of the Chi-square test results on the preprocessed dataset is shown in Appendix II. [file 9391136.f1.zip › APPENDIX-I.docx]

APPENDIX – I: Dataset Attributes

| **Sl. No.** | **Attribute** | **Type** | **Information** |
| --- | --- | --- | --- |
| 1. | donor_age | Numeric | The donor's age at hematopoietic stem cell apheresis |
| 2. | donor_age_below_35 | Boolean | Is the donor under the age of 35? |
| 3. | donor_ABO | Categorial | The hematopoietic stem cell donor's ABO blood group |
| 4. | donor_CMV | Categorial | Cytomegalovirus infection prior to transplantation in the donor of hematopoietic stem cells |
| 5. | recipient_age | Numeric | The recipient's age at hematopoietic stem cell apheresis |
| 6. | recipient_age_below_10 | Boolean | Is the recipient's age under ten? |
| 7. | recipient_age_int | Categorial | Distinct intervals of the recipient's age |
| 8. | recipient_gender | Categorial | The recipient's gender |
| 9. | recipient_body_mass | Numeric | Mass of the transplanted hematopoietic stem cell recipient |
| 10. | recipient_ABO | Categorial | The recipient's ABO blood group |
| 11. | recipient_rh | Categorial | The Rh factor is present on the recipient's red blood cells |
| 12. | recipient_CMV | Categorial | Cytomegalovirus infection prior to transplantation in the donor of hematopoietic stem cells |
| 13. | disease | Categorial | Disease classification |
| 14. | disease_group | Categorial | Malignant or nonmalignant |
| 15. | gender_match | Categorial | Gender compatibility between donor and recipient |
| 16. | ABO_match | Categorial | HSC donor-recipient blood group compatibility |
| 17. | CMV_status | Categorial | Serological compatibility of hematopoietic stem cell donors and recipients based on CMV infection prior to transplantation |
| 18. | HLA_match | Categorial | Antigen compatibility between the donor and receiver of hematopoietic stem cells |
| 19. | HLA_mismatch | Categorial | HLA mismatches or matches |
| 20. | antigen | Categorial | How many antigens differ between the donor and receiver |
| 21. | allel | Categorial | How many alleles differ between the donor and receiver |
| 22. | HLA_group_1 | Categorial | The donor-recipient difference |
| 23. | risk_group | Categorial | Group at risk |
| 24. | stem_cell_source | Categorial | Hematopoietic stem cell source |
| 25. | tx_post_relapse | Boolean | The second bone marrow transplant following recurrence |
| 26. | CD34_x1e6_per_kg | Numeric | CD34kgx10d6 - CD34+ dose of cells per kg mass of recipient |
| 27. | CD3_x1e8_per_kg | Numeric | Dose of CD3+ cells per kilogram of recipient weight |
| 28. | CD3_to_CD34_ratio | Numeric | The ratio of CD3+ cells to CD34+ cells |
| 29. | ANC_recovery | Numeric | Time required for neutrophil recovery is defined as a neutrophil count greater than 0.5 x 10^9/L |
| 30. | PLT_recovery | Numeric | Platelet reproducing period is defined as count >50000/mm3. |
| 31. | acute_GvHD_II_III_IV | Boolean | Development of stage II, III, or IV acute graft versus host disease |
| 32. | acute_GvHD_III_IV | Boolean | Stage III or IV growth of acute graft versus host disease |
| 33. | time_to_acute_GvHD_III_IV | Numeric | Time required for the onset of stage III or IV acute graft against host disease |
| 34. | extensive_chronic_GvHD | Categorial | Chronic graft versus host disease develops to a large extent |
| 35. | relapse | Boolean | Disease relapse |
| 36. | survival_time | Numeric | In days, the time of observation or time to event |
| 37. | survival_status | Categorial | Status of survival |
