# Supplementary material for: Survival Prediction of Children Undergoing Hematopoietic Stem Cell Transplantation Using Different Machine Learning Classifiers by Performing Chi-Square Test and Hyperparameter Optimization: A Retrospective Analysis
Source: Comput Math Methods Med. 2022 Sep 25;2022:9391136. doi: 10.1155/2022/9391136 (PMC9527434; doi:10.1155/2022/9391136)
Supplement: Supplementary Materials — The attributes of the dataset are listed in Appendix I, and the summary of the Chi-square test results on the preprocessed dataset is shown in Appendix II. [file 9391136.f1.zip › APPENDIX-II.docx]

APPENDIX – II: Chi-square test results.

| **Rank** | **Attribute** | **Index in the**  **original dataset** | **Chi-squared**  **Score** |
| --- | --- | --- | --- |
| 1 | PLT_recovery | 7 | 20390478.3300 |
| 2 | ANC_recovery | 6 | 5996503.1490 |
| 3 | time_to_acute_GvHD_III_IV | 8 | 425033.1475 |
| 4 | survival_time | 9 | 82924.0104 |
| 5 | recipient_body_mass | 2 | 115.2398 |
| 6 | CD34_x1e6_per_kg | 3 | 33.0659 |
| 7 | CD3_x1e8_per_kg | 4 | 30.4617 |
| 8 | CD3_to_CD34_ratio | 5 | 22.9098 |
| 9 | recipient_age | 1 | 21.8122 |
| 10 | relapse_yes | 57 | 15.2012 |
| 11 | disease_lymphoma | 26 | 10.8000 |
| 12 | extensive_chronic_GvHD_yes | 56 | 4.5430 |
| 13 | acute_GvHD_III_IV_yes | 55 | 2.3408 |
| 14 | tx_post_relapse_yes | 53 | 2.2043 |
| 15 | donor_ABO_AB | 12 | 2.1356 |
| 16 | recipient_age_below_10_yes | 15 | 1.9963 |
| 17 | recipient_age_int_10_20 | 16 | 1.9416 |
| 18 | risk_group_low | 51 | 1.5054 |
| 19 | CMV_status_1 | 31 | 1.4080 |
| 20 | donor_age | 0 | 1.3863 |
| 21 | allel_4 | 44 | 1.2000 |
| 22 | donor_age_below_35_yes | 10 | 1.0782 |
| 23 | disease_group_nonmalignant | 28 | 0.8167 |
| 24 | disease_nonmalignant | 27 | 0.8167 |
| 25 | donor_ABO_A | 11 | 0.7892 |
| 26 | HLA_group_1_three_diffs | 49 | 0.6750 |
| 27 | stem_cell_source_peripheral_blood | 52 | 0.6703 |
| 28 | recipient_rh_plus | 22 | 0.4602 |
| 29 | HLA_match_10-Jul | 34 | 0.4267 |
| 30 | HLA_group_1_mismatched | 46 | 0.4267 |
| 31 | antigen_1 | 38 | 0.4063 |
| 32 | antigen_3 | 40 | 0.3857 |
| 33 | recipient_age_int_5_10 | 17 | 0.3765 |
| 34 | acute_GvHD_II_III_IV_yes | 54 | 0.3440 |
| 35 | ABO_match_mismatched | 30 | 0.3380 |
| 36 | recipient_ABO_A | 19 | 0.3197 |
| 37 | donor_CMV_present | 14 | 0.2630 |
| 38 | recipient_ABO_AB | 20 | 0.2564 |
| 39 | CMV_status_3 | 33 | 0.1984 |
| 40 | disease_chronic | 25 | 0.1896 |
| 41 | recipient_CMV_present | 23 | 0.1361 |
| 42 | HLA_match_10-Sep | 36 | 0.1313 |
| 43 | HLA_match_10-Oct | 35 | 0.1280 |
| 44 | HLA_group_1_matched | 45 | 0.1280 |
| 45 | HLA_group_1_one_antigen | 48 | 0.0794 |
| 46 | donor_ABO_B | 13 | 0.0762 |
| 47 | allel_1 | 41 | 0.0733 |
| 48 | antigen_2 | 39 | 0.0611 |
| 49 | allel_3 | 43 | 0.0500 |
| 50 | recipient_gender_male | 18 | 0.0429 |
| 51 | recipient_ABO_B | 21 | 0.0427 |
| 52 | HLA_group_1_one_allel | 47 | 0.0381 |
| 53 | allel_2 | 42 | 0.0375 |
| 54 | CMV_status_2 | 32 | 0.0356 |
| 55 | HLA_group_1_two_diffs | 50 | 0.0281 |
| 56 | HLA_mismatch_mismatched | 37 | 0.0107 |
| 57 | gender_match_other | 29 | 0.0054 |
| 58 | disease_AML | 24 | 0.0000 |
